# Zebrafish against cancer: towards clinical relevance

**DOI:** 10.1242/dmm.053145

**Published:** 2026-09-28

**Authors:** Gabriel Forn-Cuni, Bruno Lopes-Bastos, David M. Langenau, E. Elizabeth Patton, B. Ewa Snaar-Jagalska

**Affiliations:** ^1^ Department of Animal Sciences, Institute of Biology Leiden, Leiden University, Einsteinweg 55, 2333 CC Leiden, The Netherlands; ^2^ Molecular Pathology Unit, Massachusetts General Hospital, Charlestown, MA, USA; ^3^ Krantz Family Center for Cancer Research, Massachusetts General Hospital, Charlestown, MA 02129, USA; ^4^ Harvard Stem Cell Institute, Harvard Medical School, Cambridge, MA 02138, USA; ^5^ MRC Human Genetics Unit, Institute of Genetics and Cancer, University of Edinburgh, Western General Hospital Campus, Edinburgh EH4 2XU, UK; ^6^ Cancer Research UK Scotland Centre, Institute of Genetics and Cancer, University of Edinburgh, Edinburgh EH4 2XU, UK

**Keywords:** Zebrafish cancer models, Translational oncology, Drug screening, Patient-derived xenografts, Personalized medicine

## Abstract

Most cancer therapeutics that show preclinical promise fail in clinical trials, underscoring the need for complementary disease models. Over the past two decades, zebrafish models have matured from a tool for studying vertebrate development into a versatile platform spanning the translational cancer pipeline. Transgenic and transplantation zebrafish models have dissected fundamental mechanisms of tumor initiation and metastasis. Beyond mechanistic insight, phenotypic chemical and genetic screens provide a rapid, whole-organism filter to prioritize and repurpose therapeutic candidates, several of which have reached the clinic. Most recently, zebrafish patient-derived xenografts have begun to predict individual patient treatment responses within clinically actionable timeframes. Tracing this evolution, we argue that zebrafish models are not a replacement for other systems but earn their translational value when applied to the questions they answer best and when embedded in co-clinical workflows alongside oncology teams.

## Teaser

**Summary:** Zebrafish cancer models cover fundamental discovery to clinical decision making. In this Perspective, we argue for their integration in co-clinical workflows, complementing existing systems to improve cancer treatment.

**Figure:**
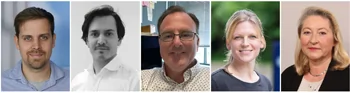
Gabriel Forn-Cuni, Bruno Lopes-Bastos, David M. Langenau, E. Elizabeth Patton and B. Ewa Snaar-Jagalska (from left to right).

## INTRODUCTION

Despite significant advances in cancer research, the incidence of cancer continues to rise, a trend that – due to population aging – is expected to continue in the following decades (Soerjomataram and Bray, 2021). In addition to fostering preventive measures, this growing burden underscores the urgent need to accelerate the discovery of effective therapies. To achieve this, it is essential to deepen our understanding of cancer biology, including the unique characteristics of each cancer type and the mechanisms underlying potential treatments (Liu et al., 2024). However, current methods for discovering new cancer therapies face significant challenges. Notably, the vast majority of drugs that show promise in preclinical studies fail to progress through clinical trials and to ultimately translate to new human therapies (Sun et al., 2022). This gap reveals the limitations of the traditional reliance on single cancer models and underscores a need for complementary approaches.

Recent advances in computational modeling (Asghar and Chung, 2025; Harkos et al., 2025) and refined *in vitro* platforms, such as patient-derived organoids and induced pluripotent stem cell (iPSC)-derived cancer models (Andersson-Rolf and Clevers, 2026; Polak et al., 2024; Tong et al., 2024), offer new avenues for exploration. In addition to this, zebrafish cancer models have emerged as a promising whole organism system for preclinical oncology. In this Perspective, we follow their journey from models of vertebrate development into a sophisticated platform that, today, underpins three pillars of cancer research: modeling human cancer to answer fundamental biology questions, screening therapeutics at scale and selecting optimal treatments for personalized medicine (Fig. 1), thus making the case for their serious consideration in translational oncology.

## A model to answer fundamental questions in cancer biology

Zebrafish were originally prized for their transparency and genetic tractability, which allowed real-time visualization of biological developmental and organogenesis processes in a living vertebrate at single-cell resolution (Kimmel et al., 1995; Streisinger et al., 1981). As genetic technologies matured – from forward mutagenesis screens to targeted transgenesis and precise CRISPR-based editing – so did the ability to faithfully recapitulate human cancers in this model. The sequencing of the zebrafish genome confirmed that >80% of disease-associated genes in humans have clear orthologs in zebrafish (Howe et al., 2013). For cancer in particular, this conservation extended from traditional oncogenes to the signaling pathways most frequently mutated in human tumors, such as RAS/MAPK, PI3K/AKT, Wnt, Notch and others.

**Fig. 1.**
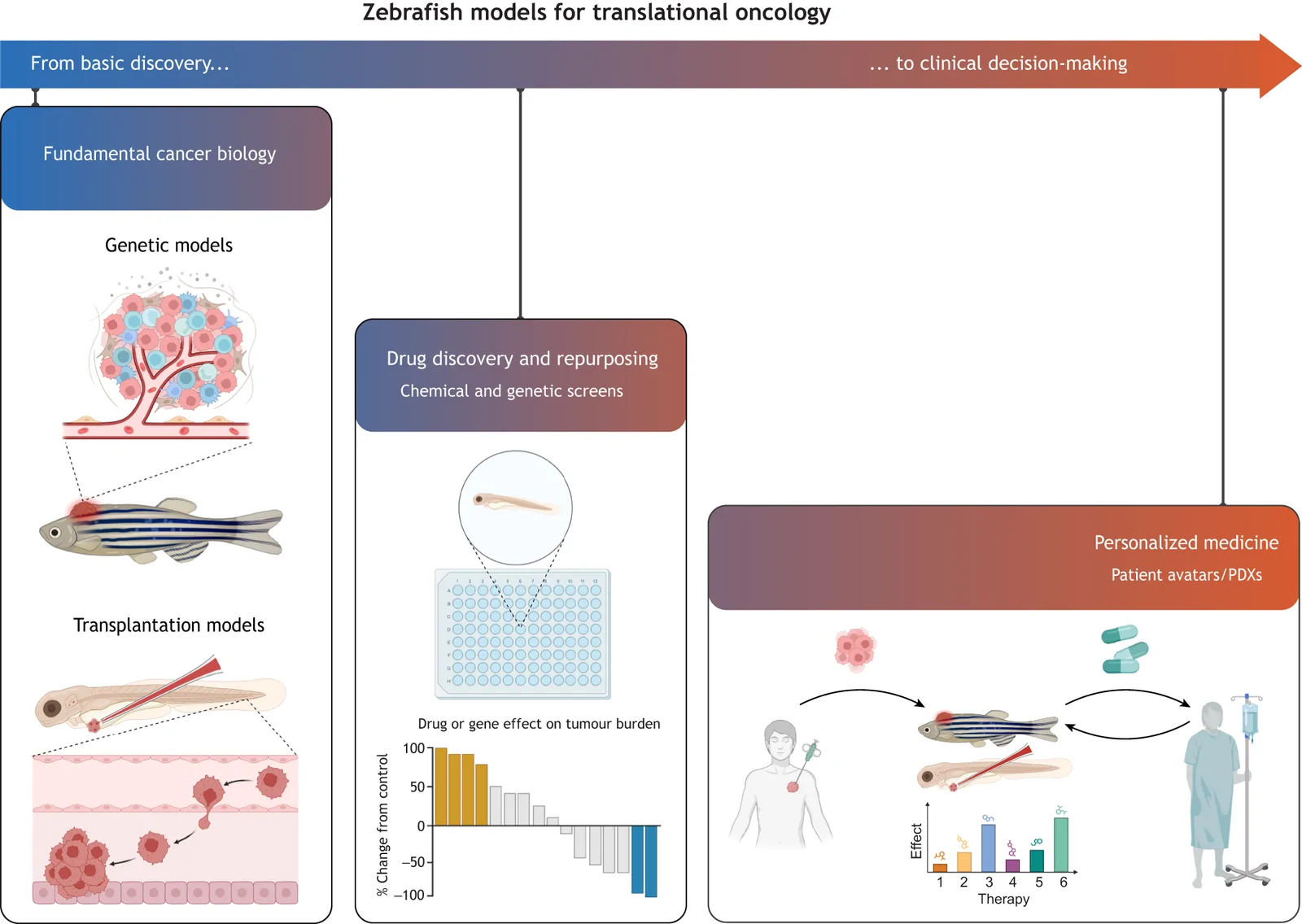
**Application of zebrafish cancer models across translational research, from basic discovery to clinical decision-making.** Fundamental biology questions can be addressed with genetic and transplantation models that dissect the mechanisms of tumor initiation, progression and metastasis. Chemical and genetic screens in larvae and adults enable rapid *in vivo* prioritization and repurposing of therapeutic candidates. Patient-derived xenografts (PDXs, or ‘avatars’), in both larval and adult formats, are well suited to personalized drug-response prediction. Created in BioRender by Forn-Cuni, G. (2026). https://BioRender.com/prdyobm. This figure was sublicensed under CC-BY 4.0 terms.

Zebrafish cancer models have taken advantage of this conservation. Over 20 years ago, the first transgenic zebrafish model of T-cell acute lymphoblastic leukemia (T-ALL) was developed by driving the MYC oncogene under the T-cell-specific *rag2* promoter (Langenau et al., 2003). Two years later, a model of melanoma was developed by driving the most common BRAF variant (BRAF^V600E^) under the melanocyte-specific promoter of zebrafish *melanocyte inducing transcription factor a* (*mitfa*), leading to the first BRAF mutant animal model (Patton et al., 2005). Currently, over a hundred genetically engineered tumor models have been established and, although not all of them have been thoroughly explored, most of them faithfully mimic the corresponding human cancer (Amatruda et al., 2002; Kobar et al., 2021; McConnell et al., 2021; Raby et al., 2020; White et al., 2013).

These models have been used to elucidate fundamental principles of oncogenesis. For example, BRAF^V600E^ overexpression alone can generate nevi but only drives melanoma in combination with p53 loss, demonstrating the importance of cooperative oncogene activation and tumor suppressor loss (Patton et al., 2005). In neuroblastoma, zebrafish models have discovered the synergistic oncogenic potential between MYCN and ALK to mediate the survival of neuroblast hyperplasia (Zhu et al., 2012). They have also proved useful in rhabdomyosarcoma to elucidate the dominance of the RAS pathway in driving transformation and to define tumor cell hierarchies, as well as showcasing the intricate balance between cell differentiation and tumorigenic potential (Ignatius et al., 2012; Langenau et al., 2007; Storer et al., 2013). Zebrafish models have clarified the mechanistic origins of cancers that previously lacked an explanation, for instance revealing the importance of SPRED1 loss in mucosal melanoma (Ablain et al., 2018). More recently, a genetic model of Ewing sarcoma has provided the first *in vivo* evidence that cells of neural crest origin uniquely tolerate the EWSR1::FLI1 fusion oncogene and may serve as the cell of origin of this pediatric cancer (Vasileva et al., 2025).

Complementing genetically engineered models, zebrafish cancer transplantation models have also been successfully applied to answer fundamental questions about cancer biology (Astell and Sieger, 2020). In these approaches, cancer cells are transplanted into zebrafish hosts, enabling the rapid *in vivo* study of tumor behavior. These transplants may be allogenic (that is, between zebrafish) or xenogeneic, most commonly using human or mouse cells. In addition, transplants may be performed orthotopically, i.e. when the zebrafish has an equivalent tissue, or heterotopically, e.g. into the circulation to study the metastatic process. To prevent rejection, most transplantations are carried out in larval stages, when the adaptive immunity of zebrafish is still immature. In addition, allogenic transplants can be transferred between syngeneic (i.e.genetically identical) fish with matched immune systems, and immunosuppressed and immunodeficient lines have been developed to allow xenografting in adult fish (Yan et al., 2020).

Transplantation models have proved ideal to explore the interaction of cancer cells with the tumor microenvironment (TME) and in metastatic processes (Weiss et al., 2022). For example, orthotopic and heterotopic allogenic transplants have been used to interrogate which TME signals establish niches that promote tumor cell dissemination (Kim et al., 2017), and how distinct transcriptional cancer states cooperate to facilitate extravasation (Campbell et al., 2021). Syngeneic transplantation in adult zebrafish has been used to follow clonal diversity in the context of stem cell potential and therapy responses, most notably in T-ALL (Smith et al., 2010) and rhabdomyosarcoma (Smith et al., 2010; Hayes et al., 2018; Ignatius et al., 2012). Finally, larval heterotopic xenografts have exhibited which hemodynamic flow forces govern the arrest, adhesion and extravasation of tumor cells in the circulation (Follain et al., 2018), and have demonstrated the important role of immune cell types, such as macrophages and neutrophils, in metastasis and angiogenesis (He et al., 2012; Yin et al., 2024).

…across tumor types driven by genetically conserved pathways […] genetically engineered zebrafish models continue to generate conceptual advances about the mechanisms behind tumorigenesis…

All in all, across tumor types driven by genetically conserved pathways – such as leukemia, melanoma, sarcoma, neuroblastoma and hepatocellular carcinoma  – genetically engineered zebrafish models continue to generate conceptual advances about the mechanisms behind tumorigenesis, providing the genetic basis of oncogenic cooperation, cancer stem cell behavior or the importance of cell of origin in determining tumor identity. However, this has not proven straightforward for all cancers, even where both the tissue and the major genetic drivers are conserved. For example, pancreatic ductal adenocarcinoma is the most common pancreatic cancer in humans, and KRAS variants are found in >90% of these cases; yet zebrafish expressing oncogenic *kras* in the pancreas develop progenitor-like, poorly differentiated tumors rather than ductal adenocarcinoma (Matar et al., 2026; Park and Leach, 2018). This divergence may reflect the lack of efficient and specific promoters for the ductal compartment. Similarly, generating a genetic model of colorectal cancer remains a challenge (Lobert et al., 2016). Homozygous mutation of *apc*, present in 80% of human colorectal cancers, is embryonic lethal in zebrafish, and heterozygotes spontaneously develop tumors across the digestive tract rather than forming localized lesions (Haramis et al., 2006). This prevents the engineering of zebrafish genetic lines with the sequential genetic variants required for full colorectal carcinoma penetrance. In these cases, as well as for cancers that depend on organs or cell types without a direct zebrafish equivalent, such as mammary, prostate or lung cancers, the field has, instead, turned to xenograft cell transplantation strategies to grow human cancers in zebrafish (Amawi et al., 2021; Fontana and Van Doan, 2024; Rong et al., 2025; Wu et al., 2023). These have proven valuable to study the importance of the microenvironment, metastatic processes and therapy responses, among others.

## High-throughput screening platforms to speed up drug discovery and repurposing

Beyond their contributions to basic science, zebrafish models are particularly valuable in prioritizing candidate compounds before committing to large-scale mammalian studies. Their suitability for screening spans both genetic and chemical approaches, and both larval and adult stages. Larvae are especially powerful for high-throughput chemical screens, as their small size allows large numbers of animals to be housed and treated simultaneously at low cost, and since their permeability to small molecules dissolved in water makes drug delivery straightforward. Furthermore, up to five days post fertilization larvae are not classified as experimental animals under current regulatory frameworks in the European Union, UK and the USA. More broadly, the biological complexity of a whole organism positions zebrafish screens as an intermediate that is more physiologically informative than traditional cell culture (at ‘system’ level), yet faster, cheaper and more ethically compliant than mouse studies, thereby aligning naturally with the 3Rs principles of Replacement, Reduction and Refinement.

The roots of zebrafish as a screening platform go back to the N-ethyl-N-nitrosourea (ENU ) mutagenesis screens of the 1990s, in which laboratories by Christiane Nüsslein-Volhard and Wolfgang Driever jointly identified ∼1500 mutations in >400 genes essential for vertebrate development, collectively published as 37 papers in the landmark 1996 issue of Development (Driever et al., 1996; Haffter et al., 1996). Shortly after, waterborne treatment of zebrafish embryos with small molecules was established as a scalable whole-organism chemical screening strategy, with 1100 compounds tested to dissect the sequence and timing of vertebrate development (Peterson et al., 2000). A subsequent screen of 5000 compounds in the *gridlock* mutant, a *hey2* model of aortic coarctation, yielded two molecules that restored aortic flow by upregulating vascular endothelial growth factor (VEGF) signaling, showing that a disease phenotype can be chemically suppressed without targeting the affected gene directly (Peterson et al., 2004). Both genetic and chemical approaches were soon exploited in a cancer context. Initially, a genetic screen identified the *crash&burn* (*crb*) mutant, a loss-of-function allele of the cell-cycle transcription factor *mybl2b* (previously known as *bmyb*), which displayed genome instability and increased cancer susceptibility (Shepard et al., 2005). This mutant, in turn, became the basis for one of the first cancer-relevant pharmacological screens in zebrafish, in which a library of 16,000 compounds was screened for suppressors of the *bmyb* phenotype, yielding three hits and establishing chemical modifier screens as a tractable strategy in cancer-relevant genetic backgrounds (Stern et al., 2005).

Several compounds derived from zebrafish cancer screens have since advanced to the clinic (Patton et al., 2021). One of the first was leflunomide, a Food and Drug Administration (FDA)-approved anti-rheumatic drug identified in a screen of 2000 small molecules for its ability to suppress a neural crest transcriptional signature that is reactivated in melanoma (White et al., 2011). Leflunomide subsequently entered clinical evaluation in combination with BRAF inhibitors (ClinicalTrials.gov ID: NCT01611675). A separate screen of 4880 compounds in a fluorescent transgenic T-ALL model, scoring for the selective loss of MYC-overexpressing thymocytes, identified the antipsychotic compound perphenazine (Gutierrez et al., 2014). This drug activates the phosphatase PP2A, which induces apoptosis in leukemia cells through a mechanism entirely orthogonal to its psychiatric indication (Gutierrez et al., 2014). This screening principle has gained significant traction by testing drug combinations whose individual safety is already established. For instance, in adult immunocompromised zebrafish xenografts, the combination of cancer drugs olaparib and temozolomide proved effective against pediatric rhabdomyosarcoma, which was validated in matched mouse xenografts (Yang et al., 2025). The efficacy of this combination therapy led to expansion of inclusion criteria for a clinical trial to include adolescents with rhabdomyosarcoma (ClinicalTrials.gov ID: NCT01858168).

Beyond prioritizing the chemical and genetic perturbations aimed at the tumor cells themselves, zebrafish screens are increasingly being applied to the broader biology of cancer treatment. Because they are carried out in a whole organism, the same assays extend naturally to host-targeted therapies, such as anti-angiogenic drugs or immunotherapies that modulate the innate immune system of the patient rather than the tumor cells directly (Astin et al., 2014). Host genetic targets can also be modulated through high-throughput F0 knockout assays, or 'crispants’ (Kroll et al., 2021), as demonstrated in the context of glioblastoma (Weiss et al., 2024), but this has not yet been applied at scale in the context of cancer. To further refine this approach, initiatives to humanize zebrafish host target proteins may be relevant. Zebrafish expressing human GM-CSF, SCF and SDF1α (officially known as CSF2, KITLG and CXCL12, respectively) maintain broader subclonal representation from engrafted patient leukemias, mitigating xenograft selection bias (Rajan et al., 2020). However, immunotherapy work still relies on delivering human effector cells into the immunodeficient zebrafish, as a functionally humanized immune system remains unachieved in zebrafish (Prajapati et al., 2024).

Zebrafish models facilitate evaluation of which therapeutic to give patients and can also inform how therapies are delivered. As mentioned above, the most common route is waterborne delivery for larvae, which has been shown to correlate well with mammalian activity for many drugs. However, while pharmacokinetic–pharmacodynamic models have been developed to translate zebrafish pharmacological parameters into clinically accurate responses (van Lieshout et al., 2026; Van Wijk et al., 2019), these have not yet been applied to zebrafish larval cancer screens. To control doses and avoid false negatives, compounds can instead be injected into the circulation of larvae. For adult zebrafish, delivery routes beyond waterborne treatment include direct injection, oral gavage and voluntary consumption of drug-infused food pellets (Dang et al., 2016; Lu and Patton, 2022). Injection into the circulation, in turn, allows the screen of different therapy formulations, such as encapsulation in liposomal carriers or lipid nanoparticles. In transparent larvae, their biodistribution, tumor targeting and cellular uptake can be followed in real time in a living vertebrate, a fact that has proved particularly useful for RNA therapeutics (Yang et al., 2017; Zhao et al., 2025).

Building on these foundations, the field is scaling these capabilities further through automation. For example, high-content imaging platforms enable automated longitudinal single-cell readouts of tumor burden in standardized 96-well xenograft assays (Sturtzel et al., 2023).

…the combination of whole-organism complexity, high-throughput capabilities, low cost and favorable regulatory standing, positions zebrafish as an efficient *in vivo* filter in the drug discovery pipeline.

Overall, the combination of whole-organism complexity, high-throughput capabilities, low cost and favorable regulatory standing, positions zebrafish as an efficient *in vivo* filter in the drug discovery pipeline. By identifying candidate therapies before they reach mammalian testing, these screens address one of the most inefficient and costly steps in translational oncology. They have proved especially powerful for repurposing approved drugs and testing combinations in new oncological contexts, bypassing the early safety evaluation required for novel compounds while allowing direct translation from zebrafish to human and guarding against species-specific false positives.

## Use in developing personalized medicine

Transgenic zebrafish models carrying patient-relevant mutations have long been used to understand cancer biology, as discussed above for BRAF^V600E^ and SPRED1. Modeling the SHP2^D61G^ mutation, for instance, revealed a pro-inflammatory signature in hematopoietic cells, nominating inflammation as a therapeutic target in juvenile myelomonocytic leukemia (Solman et al., 2022). The same approach has recently uncovered the role of nuclear RNA surveillance as a tumor-suppressive pathway. Recapitulating patient CDK13 mutations drives melanoma in zebrafish by stabilizing aberrant protein-coding transcripts (Insco et al., 2023), and truncating mutations in the surveillance factor ZC3H18 that is lost in ∼30% of cancers, accelerated melanoma onset in zebrafish by stabilizing endogenous retroviral RNA (Tanu et al., 2026).

However, these genetic models remain mostly preclinical tools: the weeks-to-months of tumor latency in addition to the time required for sequencing and vector construction, place them outside the window of most clinical decisions. The more immediate clinical application comes instead from zebrafish patient-derived xenograft (PDX) models, also known as patient avatars (Fazio et al., 2020). In these models, tumor cells of a patient are engrafted into zebrafish and exposed to candidate therapies to predict which one will work best for that individual.

Larval PDXs are particularly well suited to this task. A small biopsy from a patient provides enough material to generate large cohorts of fluorescently labeled xenografts, which can be treated with candidate drugs and provide results within roughly a week. Drug responses in larval PDXs from a range of cancer origins have consistently correlated with patient clinical responses, including tests for multiple myeloma (Lin et al., 2016), breast cancer (Mercatali et al., 2016), colorectal cancer (Fior et al., 2017), B-cell precursor acute lymphoblastic leukemia (Gauert et al., 2020), gastric cancer (Zhai et al., 2021), non-small cell lung cancer (Hua et al., 2023), urinary bladder cancer (Kowald et al., 2023) and ovarian cancer (Lindahl et al., 2024).

As a consequence, this kind of personalized testing has been formalized in co-clinical trials evaluating the predictive power of zebrafish PDXs across several cancer types, including gastrointestinal solid cancers (Usai et al., 2020), pancreatic ductal adenocarcinoma (Usai et al., 2021), colorectal cancer (Di Franco et al., 2022) and non-small cell lung cancer (Ali et al., 2022). In matched cohorts, zebrafish patient avatar tests have reproduced patient responses in 50 of 55 patients with colorectal cancer (Costa et al., 2024), in all 18 patients of a breast cancer cohort (Mendes et al., 2025) and in 29 out of 32 patients with ovarian cancer (Estrada et al., 2026). Importantly, the timeframe of these models is compatible with clinical decision-making, as recently highlighted by a report of zebrafish patient avatars used in a case of recurrent gliosarcoma, a rare and aggressive glioblastoma subtype for which treatment options after relapse are scarce and lack reliable biomarkers (Fontes et al., 2025). The zebrafish PDXs identified the most effective drug combinations and recapitulated the acquired resistance of the patient to previously failed therapies, guiding treatment choices across successive relapses in close collaboration with clinicians.

…zebrafish PDXs are fast and accurate enough to assess real treatment options for an individual patient.

Taken together, these results demonstrate that zebrafish PDXs are fast and accurate enough to assess real treatment options for an individual patient. However, this use of PDXs to assist clinicians in guiding personalized treatment selection, while promising, is still being developed at a limited scale. Given that many laboratories routinely use zebrafish PDXs, the reason may be logistical. Ranking candidate drugs for an individual patient within a short decision window requires infrastructure embedded alongside the clinic, with established routes for fresh patient material, hospital-available drugs, and constant interaction between researchers and clinicians.

## Closing words

Over the past two decades, zebrafish cancer models have expanded from a system used to study vertebrate development into clinically relevant actors in the translational pipeline. Building on a unique combination of properties, they have been used to dissect the fundamental biology of cancer, to discover and prioritize therapeutic compounds and, most recently, to test treatment options for individual patients. In this Perspective, we have followed this progression to show how zebrafish cancer models can contribute to every stage of translational oncology, from basic discovery to clinical decision-making.

The value of differing zebrafish models emerges when they are applied to the questions they answer best – as one complementary system within a broader toolkit. To achieve optimal clinical translation, zebrafish research must stay directed to medically relevant questions; this requires sustained collaboration with clinicians and patients, who can ensure that experimental endpoints correspond to meaningful outcomes.

But zebrafish cancer models do have some limitations (Cabezas-Sáinz et al., 2020). Adults can be maintained at 37°C for the duration of a xenografting experiment, but larval xenografts are kept at a compromise temperature of 33−35°C that is suboptimal for either the developing zebrafish or the xenografted tumor cells. In addition, working with a model that is undergoing embryonic development can affect therapy responses or restrict the experimental window for tissues that mature later. Furthermore, several of the properties that make the PDX larval model powerful for personalized medicine also determine its constraints. For example, the short experimental duration is a double-edged sword. While it delivers answers in days, it cannot capture recurrence or resistance over the timescale of patient treatment. Similarly, the immature adaptive immunity that permits xenografting without rejection also prevents testing approaches that depend on endogenous T-cell responses, such as checkpoint blockade.

In that sense, it is important to highlight that zebrafish models are not a replacement for other systems. Mammalian models, such as mouse models, remain necessary for late-stage validation, organoids offer higher patient cellular fidelity *in vitro* and computational methods address questions no *in vivo* model can, such as the exploration of chemical space that has never been synthesized and of evolutionary dynamics over big timescales. The value of differing zebrafish models emerges when they are applied to the questions they answer best – as one complementary system within a broader toolkit. To achieve optimal clinical translation, zebrafish research must stay directed to medically relevant questions; this requires sustained collaboration with clinicians and patients, who can ensure that experimental endpoints correspond to meaningful outcomes (Amatruda, 2021; Amatruda, 2023). In an integrated setting among other models and in collaboration with clinicians and patients, zebrafish models are positioned to earn an impactful role in the fight against cancer.
